# A Rare Case of COVID-19 Presenting as Acalculous Cholecystitis

**DOI:** 10.7759/cureus.46332

**Published:** 2023-10-01

**Authors:** Nayaab Bakshi, Nibras Yar Khan, Navjot Grewal, Alaa Muhanna, Jihad Slim

**Affiliations:** 1 Internal Medicine, Saint Michael's Medical Center, Newark, USA; 2 Infectious Diseases, Saint Michael's Medical Center, Newark, USA

**Keywords:** sars-cov-2 complications, sars-cov-2, covid 19, cholecystitis, acalculous cholecystitis

## Abstract

At its onset, coronavirus disease 2019 (COVID-19) commonly presents with generalized myalgia and upper respiratory symptoms. COVID-19 presenting as acalculous cholecystitis has been rarely described in the literature. The following case presents a patient whose first presentation of COVID-19 was acalculous cholecystitis without respiratory symptoms, critical illness, or severe COVID-19 infection.

## Introduction

Coronavirus disease 2019 (COVID-19) is an infectious disease caused by the respiratory virus severe acute respiratory syndrome coronavirus 2 (SARS‑CoV‑2). Over 760 million cases and 6.8 million deaths have been recorded to date globally [1]. COVID-19 typically presents with cough, myalgias, headache, sore throat, and taste or smell abnormalities. Up to 26% of patients diagnosed with COVID-19 have gastrointestinal symptoms [2]. Among gastrointestinal symptoms, gallbladder involvement is extremely rare. Though COVID-19 carries a high mortality rate, gastrointestinal complications are not one of the top 10 causes of COVID-19-associated deaths [3].

Acute acalculous cholecystitis is the acute inflammation of the gallbladder in the absence of stones or cystic duct obstruction [4]. It comprises 10% of acute cholecystitis cases and is most commonly observed in the setting of critically ill patients who often require ICU and mechanical ventilation [5]. Interestingly, studies show that only 10% of patients hospitalized with COVID-19 require intensive care [6].

Therefore, a patient with COVID-19 presenting as acalculous cholecystitis, a condition itself known to have a mortality of 30-50%, without initial accompanying respiratory symptoms or critical illness, is extremely rare [7]. Herein, we report such a case.

## Case presentation

A 75-year-old female with a past medical history of hypertension, hyperlipidemia, non-insulin-dependent diabetes mellitus, anterior cervical spine discectomy and fusion, right-sided nephrolithiasis, and gastroesophageal reflux disease presented to the emergency department complaining of sudden-onset right upper quadrant pain for one day.

The pain was sharp and continuous, radiating to her right flank in a band-like manner. It was associated with nausea and multiple episodes of non-bloody and yellow-colored vomiting. There were no known aggravating or alleviating factors. The patient had never had similar episodes in the past. The patient denied fever, chills, dizziness, rhinorrhea, congestion, myalgias, chest pain, dyspnea, cough, diarrhea, constipation, hematemesis, hematochezia, dysuria, hematuria, weakness, numbness, or tingling. The patient admitted to living with her grandchildren, who had been feeling unwell with upper respiratory symptoms. There was no history of recent infections or antibiotic use. The patient was afebrile and hemodynamically stable, saturating at 98% on room air.

On physical examination, the patient did not have any jaundice, scleral icterus, or rashes. Auscultation of the abdomen revealed normoactive bowel sounds. Murphy’s sign (tenderness to palpation of the right upper quadrant on deep inspiration) was positive and the patient's right upper abdominal quadrant, bilateral lower abdominal quadrants, and right flank were tender to palpation. Cardiac examination revealed regular rate and rhythm, and pulmonary exam revealed fair air entry and lungs clear to auscultation bilaterally without wheezes, rales, or rhonchi.

Labs showed that white blood cells were 12,500/microliters, C-reactive protein was 11.5 milligrams/liter, and lactate dehydrogenase was 224.2 units/liter. Aspartate aminotransferase (AST), alanine transaminase (ALT), and alkaline phosphatase (ALP) were 30, 39, and 85 units/liter respectively. Total bilirubin was 1.0.

**Figure 1:**
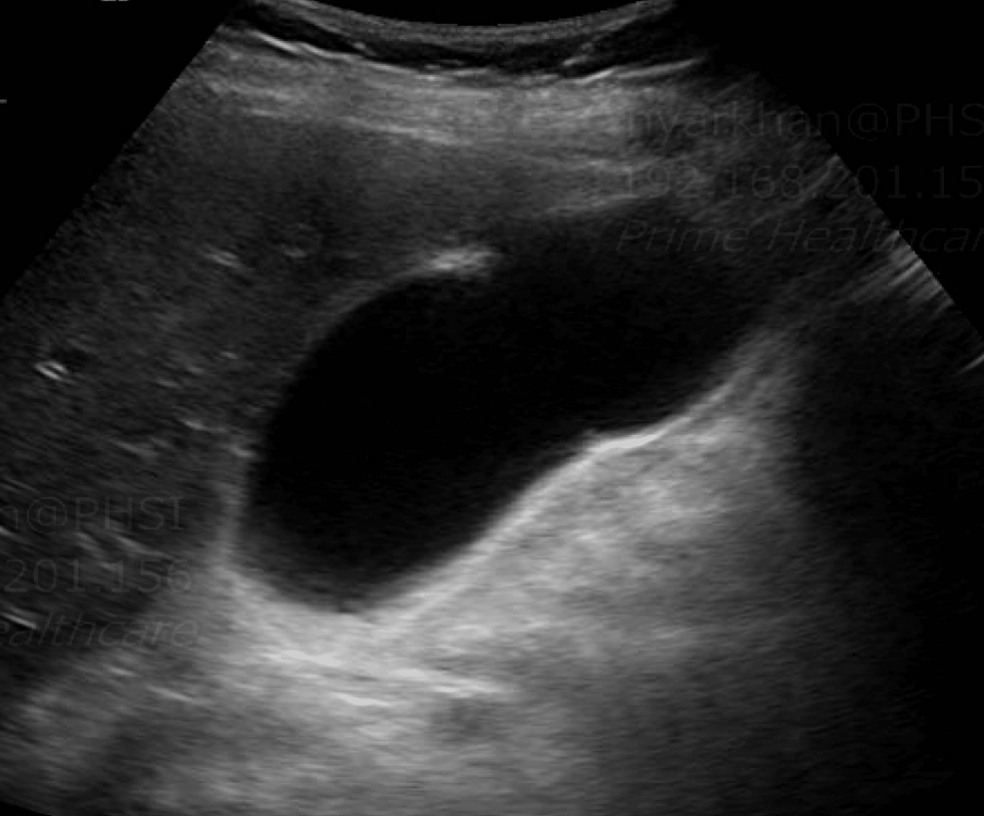
Ultrasound showed gallbladder distension and pericholecystic fluid but no evidence of gallstones

**Figure 2:**
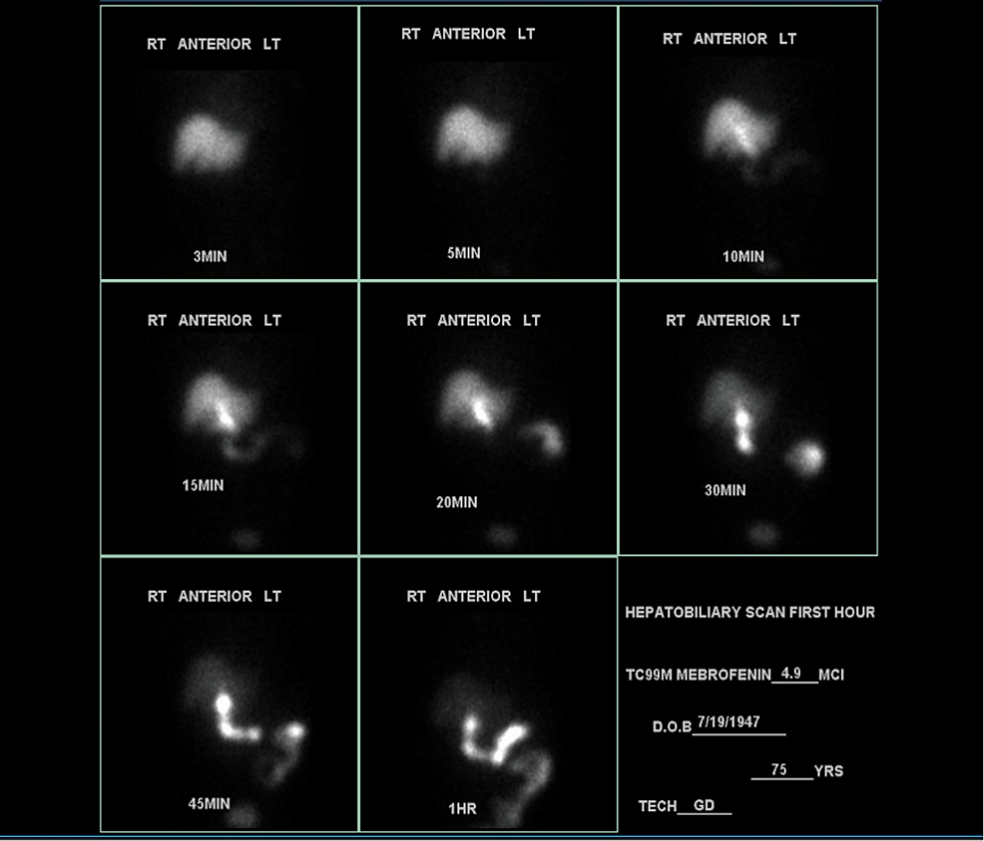
Hepatobiliary iminodiacetic acid (HIDA) scan showed cystic duct obstruction

COVID antigen was positive, polymerase chain reaction (PCR) was negative, and rapid influenza was negative. The CT of the abdomen and pelvis showed gallbladder distention with likely a small amount of pericholecystic fluid. Left nephrolithiasis without evidence of obstruction, and colonic diverticulosis without evidence of diverticulitis, were also found. The patient was given ceftriaxone and metronidazole. The gallbladder on ultrasound showed minimal pericholecystic fluid but no evidence of gallstones or a sonographic Murphy's sign (Figure 1). Given the high suspicion of cholecystitis, a hepatobiliary iminodiacetic acid (HIDA) scan was ordered, which revealed cystic duct obstruction (Figure 2). Surgery was consulted, and laparoscopic cholecystectomy was performed. The surgical specimen collected for pathology indicated acute cholecystitis with focally hemorrhagic and necrotic mucosa with adherent necrotic mucoid tissue without gallstones.

**Figure 3:**
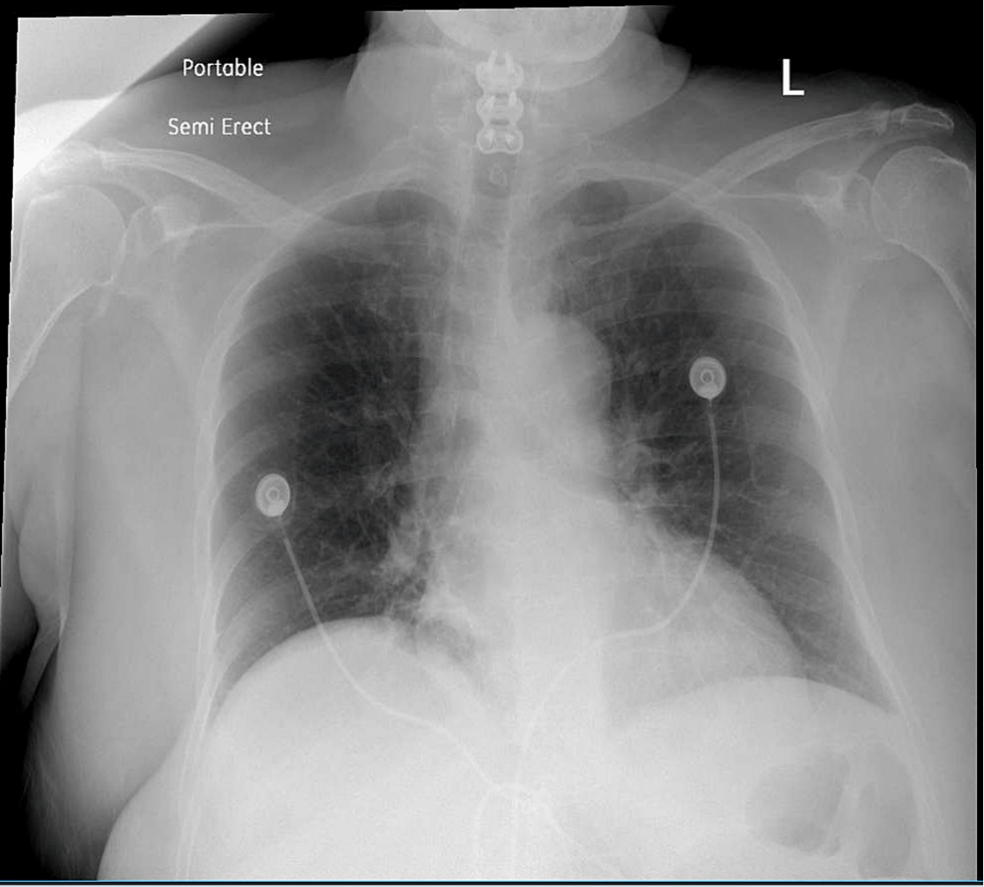
Chest X-ray Chest X-ray demonstrated mild elevation of the right hemidiaphragm. The lungs were clear. No pneumothorax or pleural effusion was identified. The cardio-mediastinal silhouette was within normal limits. No aggressive osseous lesions were seen.

On postoperative day one, the patient’s pain was minimal, and she was able to tolerate a regular diet. The patient developed a productive cough with sputum but denied additional systemic symptoms. A chest X-ray was ordered, which was negative for any acute cardiopulmonary disease (Figure 3). Molnupiravir was initiated.

## Discussion

The relatively stable, non-critically ill patient was diagnosed with acute acalculous cholecystitis, which was likely caused by COVID-19 infection, given the lack of other serious comorbidities and acute illnesses generally implicated in acute acalculous cholecystitis. Though the COVID-19 antigen was positive and the COVID-19 PCR was negative, it is likely that the COVID-19 PCR was a false negative, given that the patient developed respiratory symptoms during the hospital course and had recent exposure to her grandchildren who were sick with upper respiratory symptoms. There was no other possible cause for the patient to have acalculous cholecystitis.

According to National Institute of Health (NIH) guidelines on the severity of COVID-19, the patient initially presented as asymptomatic because the patient tested positive for COVID but had “no symptoms consistent with COVID-19.” Later, the patient was classified as having a mild illness, as she had a productive cough but did not have the typical findings of “shortness of breath, dyspnea, or abnormal chest imaging” [8]. Upon literature review, we found very few cases of non-critically ill patients with COVID-19 infections without pneumonia with initial presentation of acalculous cholecystitis [9,10]. Our patient is unique because even in the rare cases reports of patients who developed acalculous cholecystitis in the setting of COVID-19, most patients had severe COVID illness, unlike our patient [11].

The mechanism of COVID-19 causing acalculous cholecystitis has been investigated. Coronavirus has a tropism for angiotensin-converting enzyme 2 (ACE2) receptors, which are abundant in the epithelium of the gallbladder. The virus enters the cells through interaction with the ACE2 receptor, and the virus has been isolated from previously reported cases of COVID-19 presenting as acute acalculous cholecystitis [9]. The exact mechanism is unknown, but “pathologically, in patients with acalculous cholecystitis, endothelial injury, gallbladder ischemia, and stasis lead to concentration of bile salts, gallbladder distension, and eventually necrosis of the gallbladder tissue” [12].

## Conclusions

COVID-19, therefore, can rarely initially present as acute acalculous cholecystitis without any other respiratory symptoms. Therefore, if a patient presents with acalculous cholecystitis, COVID-19 should be considered as a differential diagnosis even if the patient has no respiratory involvement and is not critically ill.
